# Multi-Omics Characterizes the Effects and Mechanisms of CD1d in Nonalcoholic Fatty Liver Disease Development

**DOI:** 10.3389/fcell.2022.830702

**Published:** 2022-04-08

**Authors:** Qiuxian Zheng, Chen Xue, Xinyu Gu, Dandan Shan, Qingfei Chu, Jing Wang, Haihong Zhu, Zhi Chen

**Affiliations:** State Key Laboratory for Diagnosis and Treatment of Infectious Diseases, National Clinical Research Center for Infectious Diseases, National Medical Center for Infectious Diseases, Collaborative Innovation Center for Diagnosis and Treatment of Infectious Diseases, The First Affiliated Hospital, Zhejiang University School of Medicine, Hangzhou, China

**Keywords:** multi-omics, mass spectrometry, transcriptomic, metabolomics, microbes

## Abstract

Nonalcoholic fatty liver disease (NAFLD) is a class of metabolic-associated liver diseases. Aberrant lipid consumption plays an important role in NAFLD pathogenesis. It has been shown CD1d can bind to multiple different lysophospholipids and associated with NAFLD progression. However, the mechanism of CD1d regulation in NAFLD is not completely understood. In this study, we established a NAFLD mouse model by feeding C57/BL6J mice a high-fat diet (HFD) for 24 weeks. Subsequently, we performed integrated transcriptomics and metabolomics analyses to thoroughly probe the role of CD1d in NAFLD progression. In the present study, we demonstrate that CD1d expression was significantly decreased in our murine model of NAFLD. Additionally, we show CD1d knockdown (CD1d KO) in HFD-fed wild-type (WT) mice induced NAFLD, which resulted in weight gain, exaggerated liver injury, and hepatic steatosis. We uncover the crucial roles of CD1d deficiency results in accumulated lipid accumulation. We further explored the CD1d deficiency in NAFLD regarding the transcriptional landscapes, microbiota environment, metabolomics change, and transcriptomics differences. In conclusion, our data demonstrate CD1d plays an important role in NAFLD pathogenesis and may represent a potential therapeutic target for the further therapy.

## Introduction

Fatty liver disease is a common disease that affects approximately 25% of the global adult population and is one of the major causes of both hepatocellular carcinoma and liver cryptogenic cirrhosis (Younossi et al., 2016). Metabolic dysfunction–associated fatty liver disease is also known as nonalcoholic fatty liver disease (NAFLD) (Zhou et al., 2019; Zeigerer, 2021). NAFLD is characterized by excessive fat accumulation, and its prevalence in China has increased to approximately 29% (Zhou et al., 2019). NAFLD is defined by excessive hepatic liver accumulation and metabolic dysregulations that are not associated with heavy alcohol consumption and meet at least one of three indicators: overweight/obesity, presence of type 2 diabetes mellitus, or clinically evident metabolic dysregulation (Eslam et al., 2020; Eslam et al., 2021). NAFLD encompasses a broad spectrum of hepatic pathologies, ranging from simple liver cell steatosis to nonalcoholic steatohepatitis (NASH). Additionally, NAFLD progression may lead to cirrhosis and ultimately hepatocellular carcinoma (Meroni et al., 2020; Chen V. L. et al., 2021). NAFLD has reached epidemic proportions and, over the next decade, may become the leading cause of cirrhosis, which requires liver transplantation (Maurice and Manousou, 2018). However, NAFLD is a complicated and multi-faceted metabolic disease involving genetic and environmental factors, such as inflammation, increased oxidative stress, mitochondrial dysfunction, and intestinal dysbiosis (Jin et al., 2021; Van Kleef et al., 2021). Moreover, the molecular mechanisms responsible for regulating NAFLD progression from a “safe” state, such as simple stenosis, to hepatitis are unclear. Fat accumulation may cause chronic hepatic steatosis, which can trigger low-grade inflammatory responses (Cuevas-Zuviría et al., 2020; Zhang et al., 2021). Low-grade inflammation may play a key role in liver steatosis initiation and progression to steatohepatitis stages (Friedman et al., 2018). It is crucial to intended to develop emerging targets that arrest or reverse disease progression (Friedman et al., 2018; Zhang et al., 2021). It is essential to identify potential therapeutic targets that arrest or reverse NAFLD progression.

Recent studies have demonstrated CD1d is involved in hepatic cell lipid deposition and liver inflammation (Cuevas-Zuviría et al., 2020; Govindarajan et al., 2020). Furthermore, recent evidence suggests CD1d may play a critical role in NAFLD development and progression (Brigl and Brenner, 2004). CD1d is a member of the CD1 family of glycoproteins, which includes CD1a, CD1b, CD1c, CD1d, and CD1e in humans (Girardi and Zajonc, 2012). Only CD1d (CD1d1 and CD1d2) is expressed in rodents, and the mouse and human CD1d genes share greater than 95% homology (Kashiwase et al., 2003; Berkers and Ovaa, 2005).

CD1d is a well-known lipid antigen receptor expressed on antigen-presenting cells, which primarily participates in the innate immune response by presenting either microbial or endogenous glycolipid antigens to natural killer T (NKT) cells (Godfrey and Rossjohn, 2011; Van Kaer et al., 2013). Although CD1d plays an important role in regulating lipid antigen reception and presentation signaling networks (Major et al., 2006). its role in the regulation of transcriptional profiles, metabolism, and gut microbiota in NAFLD remains unclarified.

In this study, we demonstrated CD1d is functionally associated with NAFLD and potentially affects lipid metabolism–associated transcriptional profiles, metabolomic profiles, and the gut microbiome. In addition, we visualized the landscape of functional links based on multiple omics, which may provide novel insight into NAFLD progression.

## Materials and Methods

### NAFLD Model Construction

Specific pathogen–free, male inbred 6-week-old C57BL/6 mice weighing 20 ± 2 g were purchased from Weitong Lihua Experimental Technology Co., Ltd. and raised at ZheJiang University Experimental Animal Center. To model NAFLD, mice were fed a high-fat diet (HFD), containing 60 kcal% fat, 20 kcal% carbohydrates, and 20 kcal% protein (D12492, Research Diets Inc.) for 24 weeks. CD1d-knockout (KO) mice (Stock No: 008,881, The Jackson Laboratory) were purchased from Jackson Laboratory. CD1d-KO mice were on the C57BL/6 genetic background. All animals were housed in an individual ventilated cage system in the specific pathogen–free facility under a 12-h light/dark cycle at 22–24°C with unrestricted access to food and water for the duration of the experiment. Animals were maintained according to the Guidance for the Care and Use of Laboratory Animals formulated by the Ministry of Science and Technology of China. The protocol for this study was approved by the ethical committee of The First Affiliated Hospital, College of Medicine, Zhejiang University (20211204).

### Serum Chemistry Analysis

Mouse blood was sampled from the eyeball. Samples were placed at room temperature for 30 min, then centrifuged at 800 g for 20 min at 4°C. The sera were collected and stored at −20°C. The sera were packaged with dry ice for transportation. The fully automatic biochemical analyzer (Chemray 800; Rayto Life and Analytical Sciences Co., Ltd., Shenzhen, China) was applied to test serum alanine aminotransferase (ALT), aspartate aminotransferase (AST), total cholesterol (TC), high-density lipoprotein (HDL), low-density lipoprotein (LDL), triglycerides (TG), and serum cholesterol (CHO).

### Body Weight Monitoring, Biochemical Analysis, and Pathological Staining

Mice were weighed weekly at 9:00 a.m., and their body weights were recorded. After 24 weeks, HFD and normal control diet feeding, mice were sacrificed under 3% pentobarbital sodium. Blood samples were collected by extracting the eyeball. Blood was centrifuged at 3,000 rpm/min for 10 min 4°C to obtain the supernatant serum. The biochemical serum parameters ALT, AST, TG, CHO, HDL, and LDL were analyzed using an automatic biochemical analyzer. The livers were divided into pieces, which were used for histopathological and multi-omics analysis. One piece of liver tissue from each mouse was fixed in 4% paraformaldehyde and stained with hematoxylin and eosin (H&E) according to the manufacturer’s protocol. Frozen, 8-μm-thick liver tissues were sectioned and stained with Oil Red O according to standard protocols. Two samples containing 250 mg fresh liver tissue from each mouse were snap-frozen with liquid nitrogen and stored at −80°C until omics analysis. Cecal contents were harvested by gently squeezing the contents from the tissue into a sterile collection tube, and tubes were immediately placed stored in liquid nitrogen until 16 S microbiomics.

### Immunohistochemical Analysis

Immunohistochemistry was used to visualize F480 and P65 expression using a standard immunohistochemical protocol, as previously described. We performed an immunohistochemical analysis of paraffin-embedded mouse liver using F4/80 (D2S9R) XP^®^ Rabbit mAb (70076T, 1:300 dilution; CST), NF-κB p65 (L8F6) Mouse mAb (6956S, 1:400 dilution; CST).

### RNA Extraction and Validation

Total liver tissue RNA was extracted using TRizol (Life Technologies, MA, United States) reagent according to the manufacturer’s instructions. RNA purity was evaluated, and RNA was quantified using the NanoDrop 2000 spectrophotometer (Thermo Scientific). RNA integrity was assessed using the Agilent 2,100 Bioanalyzer (Agilent Technologies).

### Flow Cytometry Analysis

The cell death was stained by DAPI, the immune cells were stained by CD45 connected with FITC. All the immune cells were stained and resuspended in PBS. We applied the BD LSR Fortessa to acquire the data. Data were analyzed using FlowJo software (TreeStar).

### Transcriptome Analysis

To identify essential regulators involved in NAFLD initiation and progression, transcriptomic analyses were performed using RNA sequencing (RNA-seq). High-throughput RNA-seq was conducted by an Illumina HiSeq™ 2,500 platform. In this analysis, the reference transcriptome sequencing of 16 samples was completed, and a total of 112.03 g CleanData was obtained. All data analysis was conducted by OE Biotech Co. Ltd. (Shanghai, China).

### Functional Annotations

To further understand the biology and predict the potential functions of differentially expressed genes (DEGs), we performed gene ontology (GO) and Kyoto Encyclopedia of Genes and Genomes (KEGG) enrichment analysis. GO is divided into three levels according to functional classification. The first level contains three items: biological process, cellular component, and molecular function. The second level contains 64 items, including biological adhesion, cell, and binding. The third layer contains tens of thousands of genes used for conventional enrichment. Function from Level 1 to Level 3 is more specific and vice versa. The KEGG pathway analysis was performed on genes encoding differentially expressed proteins using KEGG database (combined with KEGG annotation results), and a hypergeometric distribution test was used to calculate the significance of the differential gene enrichment of each pathway item.

### Nontargeted Metabolomics of Liver Tissues

Metabolomics is an umbrella term that includes targeted metabolomics and untargeted metabolomics (Minhas et al., 2019). The main difference is that targeted metabolomics only examines specific metabolites. Sample preparation for metabolomic analysis has been described previously (Sailani et al., 2020; Yu et al., 2021). Untargeted metabolomics, including sample pretreatment, metabolite extraction, liquid chromatography–mass spectrometry (LC-MS) full scan detection, data pretreatment, and statistical analysis was conducted by OE Biotech (Shanghai, China). Raw data were standardized during preprocessing, and raw data were analyzed qualitatively and quantitatively based on the untargeted metabolomics of ultra-performance liquid chromatography-tandem high-resolution mass spectrometer, combined with the metabolomics data processing software Progenesis QI V2.3.

### Integrated Metabolomics and Transcriptomics Analysis

In this study, we adopted the liver tissue based transcriptome analysis to explore the difference between four groups. We also applied the non-target metabolomic omics analysis to evaluated the metabolism difference between these grouos. We calculated the *p*-value and fold change of the top 100 DEGs and metabolites and integrated the transcriptomics- and metabolomics-determined relative transcript levels of the four subgroups. The correlation between the response intensity data of genes and metabolites was calculated based on Pearson’s correlation analysis, and the network graph was drawn with a *p*-value ≤ 0.05.

### Metabolome Profiling Analysis

To comprehensively described the gut microbial difference in four subgroups, we used the metabolome profiling analysis. Feces were collected for gut microbial analysis. A total of 60 mg mouse feces from the cecum were collected under sterile conditions, placed into sterile tubes, and immediately snap-frozen in liquid nitrogen and stored at −80°C (Chen F. et al., 2021). Preparation for LC-MS analysis was performed as previously described. The fecal pellets were harvested, and total DNA was isolated according to the manufacturer’s instructions. Subsequent sequencing was conducted using the Illumina MiSeq platform (Illumina, California, United States) by OE Biotech (Shanghai, China). Raw data were converted to FASTQ formats, and the sequences were demultiplexed and quality-filtered using the QIIME (version 1.8.0) software package. Vsearch software was applied to generate operational taxonomic units (OTUs), and sequences with >97% similarity were assigned to the same OTUs. All representative reads were annotated and BLASTed using Silva database version 123 (or Greengens) with RDP classifier (confidence threshold was 70%).

### Statistical Analysis

Statistical analysis was performed using GraphPad Prism software version 7 (La Jolla, CA, USA). All animal data were presented as the mean ± standard deviation (SD). Differences between groups were assessed by one-way analysis of variance (ANOVA) or two-way ANOVA. Statistical significance was determined using an unpaired t-test analysis when comparing two groups. Significant differences were accepted at *p* < 0.05.

## Result

### CD1d Decreases in NAFLD

**FIGURE 1:**
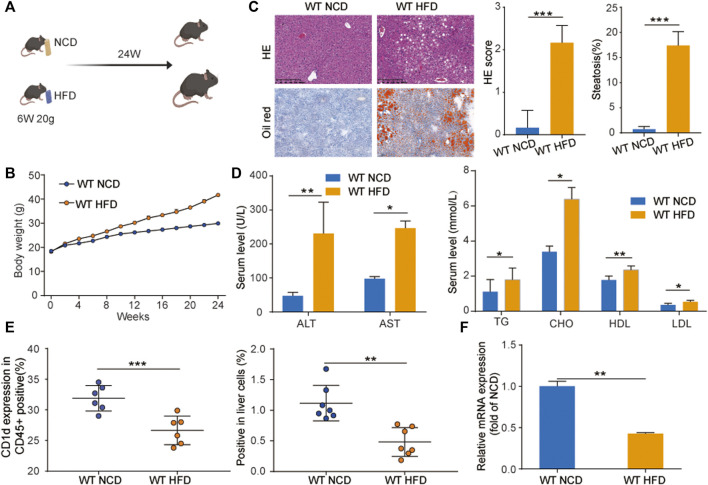
CD1d was decreased in WT-NAFLD mice. **(A)** WT mice were divided into two groups and fed with NCD and HFD separately for 24 weeks. **(B)** Body weight gain curve of WT-NCD mice and WT-HFD mice. **(C)** Liver injury and serum lipid indicators. ALT, AST, TC, TG, HDL, and LDL levels in mice serum **(D)**. The liver morphology, H&E staining and Oil Red staining, **(E)** CD1d surface marker expression in mouse CD45 ^+^ immune cells and mice liver cells. **(F) **The RNA expression level of CD1d expression between WT-NCD mice and WT-HFD mice. ALT, alanine aminotransferase; AST, aspartate aminotransferase; HDL, high-density lipoprotein; H&E, hematoxylin and eosin; HFD, high-fat diet; LDL, low-density lipoprotein; NCD, normal control diet; PBMC, peripheral blood mononuclear cell; TC, total cholesterol; TG, triglyceride; WT, wild-type.

First, we evaluated CD1d expression in our NAFLD mouse model. To develop this model, C57BL/6 mice were fed a high-fat diet (WT-HFD) or normal control diet (WT-NCD) for 24 weeks (Figure 1A) WT-HFD mice exhibited significantly faster weight gain compared to WT-NCD mice, as demonstrated by a weight growth curve (Figure 1B). We examined pathological morphology by H&E staining and Oil Red staining, which revealed significant steatosis in the livers of WT-HFD mice after 24 weeks (Figure 1C). Analysis of ALT and AST serum levels indicated WT-HFD mice had severe liver injury. Additionally, serum lipid levels were much higher in the WT-HFD group compared to WT-NCD (Figure 1D). The CD1d expression in WT HFD blood immune cells (CD45+) was significantly decreased compared with WT NCD. CD1d expression in WT HFD liver cells was significantly decreased compared with WT NCD group (Figure 1E). The mRNA expression level of CD1d in WT HFD liver tissues was remarkably decreased in WT HFD group compared with WT NCD group.

### CD1d-Deficient Mice Have Increased Body Weight Gain, Liver Injury, Hepatic Steatosis, Macrophage Cell Infiltration, and Adipocyte Volume

**FIGURE 2:**
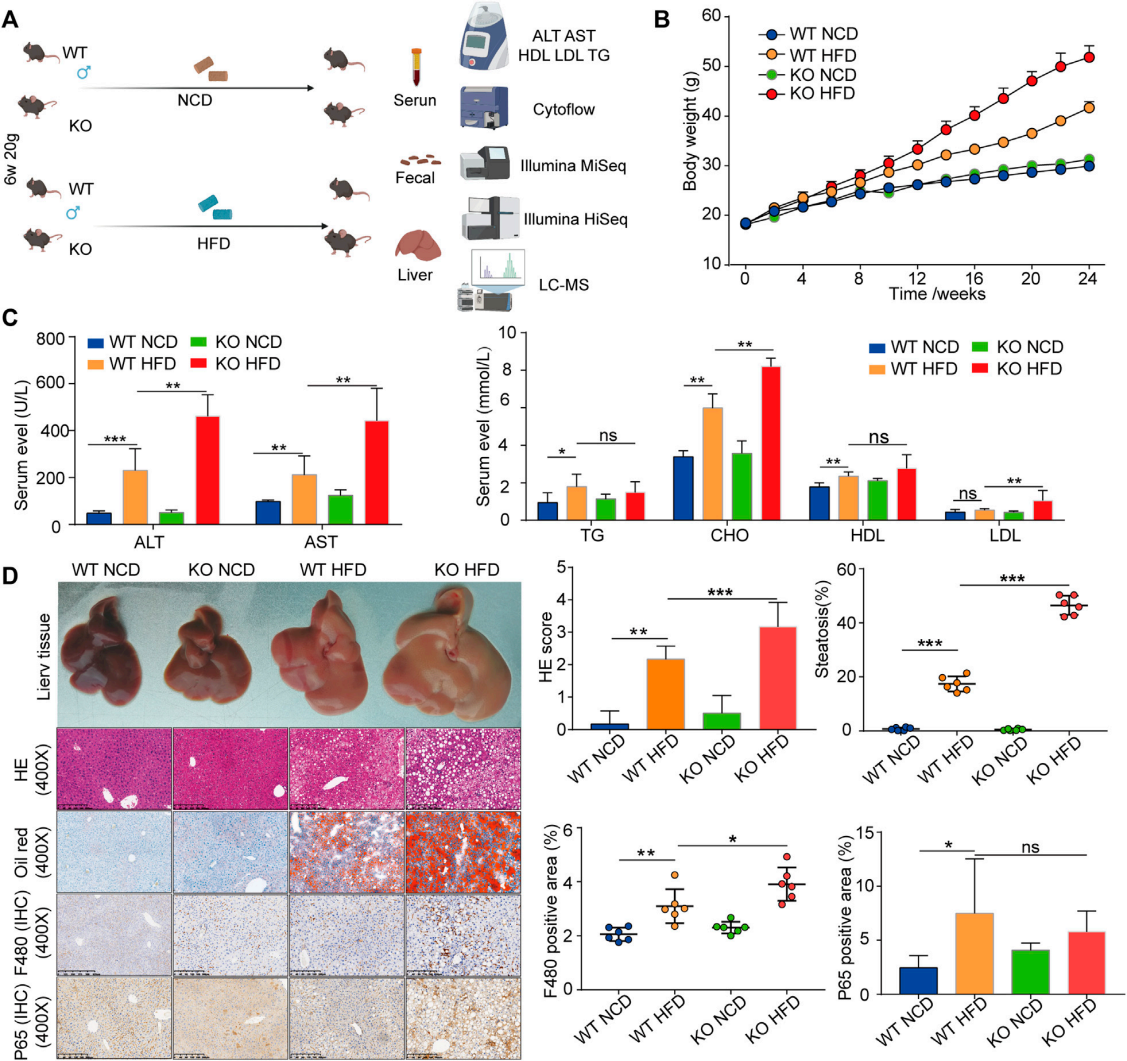
The CD1d KO mice fed an HFD display increased body weights, liver steatosis, liver injury, and fat accumulation. **(A)** WT mice and CD1d KO mice were fed HFD and NCD for 24 weeks to induce the NAFLD mouse model. **(B)** Body weight curves of four groups. **(C)** Serum ALT, ALT, TG, TC, HDL, LDL expression levels in the four groups. **(D)** Gross morphology, H&E staining, Oil Red O staining, Masson’s trichrome staining, immunohistochemistry staining of F480 and p65 of liver tissues between four groups. ALT, alanine aminotransferase; AST, aspartate aminotransferase; HDL, high-density lipoprotein; H&E, hematoxylin and eosin; HFD, high-fat diet; KO, knockout; LDL, low-density lipoprotein; NCD, normal control diet; NAFLD, nonalcoholic fatty liver disease; TC, total cholesterol; TG, triglyceride; WT, wild-type.

To investigate the role of CD1d in HFD-induced liver steatosis, we fed HFD and NCD to WT and CD1d KO mice (CD1d KO-HFD and CD1d KO-NCD, respectively) (Figure 2A). Body growth curves demonstrated that CD1d KO mice grew faster than WT mice (Figure 2B). After 24 weeks on HFD and NCD, CD1d KO-HFD mice showed severe liver injury and elevated serum lipid levels (Figure 2C). Additionally, we evaluated liver morphology, liver index, and liver pathology using H&E, Oil Red O staining, and immunohistochemistry staining for F480 and P65. in the four groups (Figure 2D). CD1d KO-HFD and WT-HFD mice exhibited increased body weight gain, liver injury, hepatic steatosis, macrophage cell infiltration, and adipocyte volume relative to WT-NCD and CD1d KO-NCD mice. These results suggest that CD1d KO-HFD mice had increased lipid accumulation and deposition.

### Identification of DEGs

**FIGURE 3:**
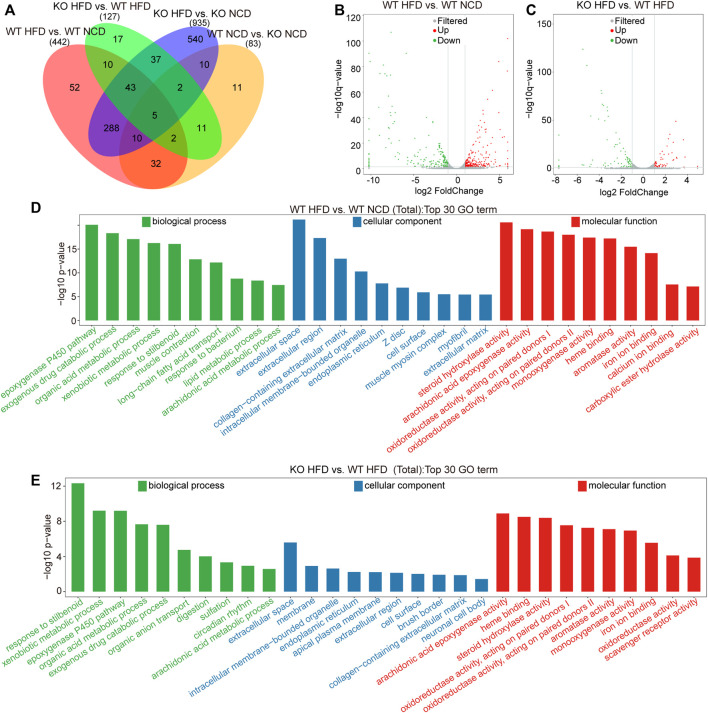
Transcriptome analysis of the liver tissues of the four subgroups. **(A)** Comparative analysis of differentially expressed genes among four groups. **(B)** Volcano plot of differential genes between WT-HFD mice and WT-NCD mice. The gray represented for non-significant difference genes, red and green are significant difference genes. **(C)** Volcano plot of differential genes between CD1d KO^−^HFD mice and WT-HFD mice. **(D)** The top 30 significantly enriched GO annotations between WT-HFD and WT-NCD mice. **(E)** The top 30 significantly enriched GO annotations between CD1d KO-HFD and WT-HFD mice. GO, gene ontology; HFD, high-fat diet; KO, knockout; NCD, normal control diet; WT, wild-type.

We applied the DESeq2 software to evaluate DEGs in the four subgroups. In this study, we identified 83 DEGs in comparisons between WT NCD and KO NCD subgroups, 442 DEGs between WT-HFD and WT-NCD group, 935 DEGs between CD1d KO-HFD and CD1d KO NCD, and 127 DEGs between CD1d KO-HFD and WT HFD (Figure 3A). The volcano plots of the DEGs identified between the WT-HFD and WT-NCD groups and between the CD1d KO-HFD and WT-HFD groups are illustrated in Figures 3B,C. The GO enrichment analysis of DEGs between the WT-HFD and WT-NCD groups revealed several enriched pathways, including muscle construction, exogenous drug catabolic process, ep-oxygenase p450 pathway, extracellular space Z disk, oxidoreductase activity, monooxygenase activity, and steroid hydroxylase activity (Figure 3D). Comparing between the WT-HFD and CD1d KO-HFD groups, DEGs were mainly enriched in the stilbenoid, xenobiotic metabolic process, extracellular space, arachidonic acid ep-oxygenase activity heme binding, and steroid hydroxylase activity pathways (Figure 3E). KEGG pathway analysis for pathways containing at least 2 DEGs was performed, and the top 20 pathways for each category were selected. KEGG analysis comparing the WT-HFD and WT-NCD groups demonstrated that DEGs were enriched in pancreatic secretion, retinol metabolism, peroxisome-proliferator-activated receptor signaling pathways, chemical carcinogenesis, fat digestion and absorption, and steroid bile acid biosynthesis (Supplementary Figure S1A). Comparing the CD1d KO-HFD and WT-HFD groups, DEGs were mainly enriched in retinol metabolism, steroid hormone biosynthesis, arachidonic acid metabolism, and chemical carcinogenesis (Supplementary Figure S1B). These studies illustrated retinol metabolism, steroid hormone biosynthesis, arachidonic acid metabolism, and chemical carcinogenesis functional pathways were active in CD1d KO-HFD. These studies suggest feeding CD1d KO mice an HFD led to accumulated aberrant lipid metabolism in the liver through retinol metabolism and steroid hormone biosynthesis process activation.

### Metabolomic Analysis of CD1d in NAFLD Progression

**FIGURE 4:**
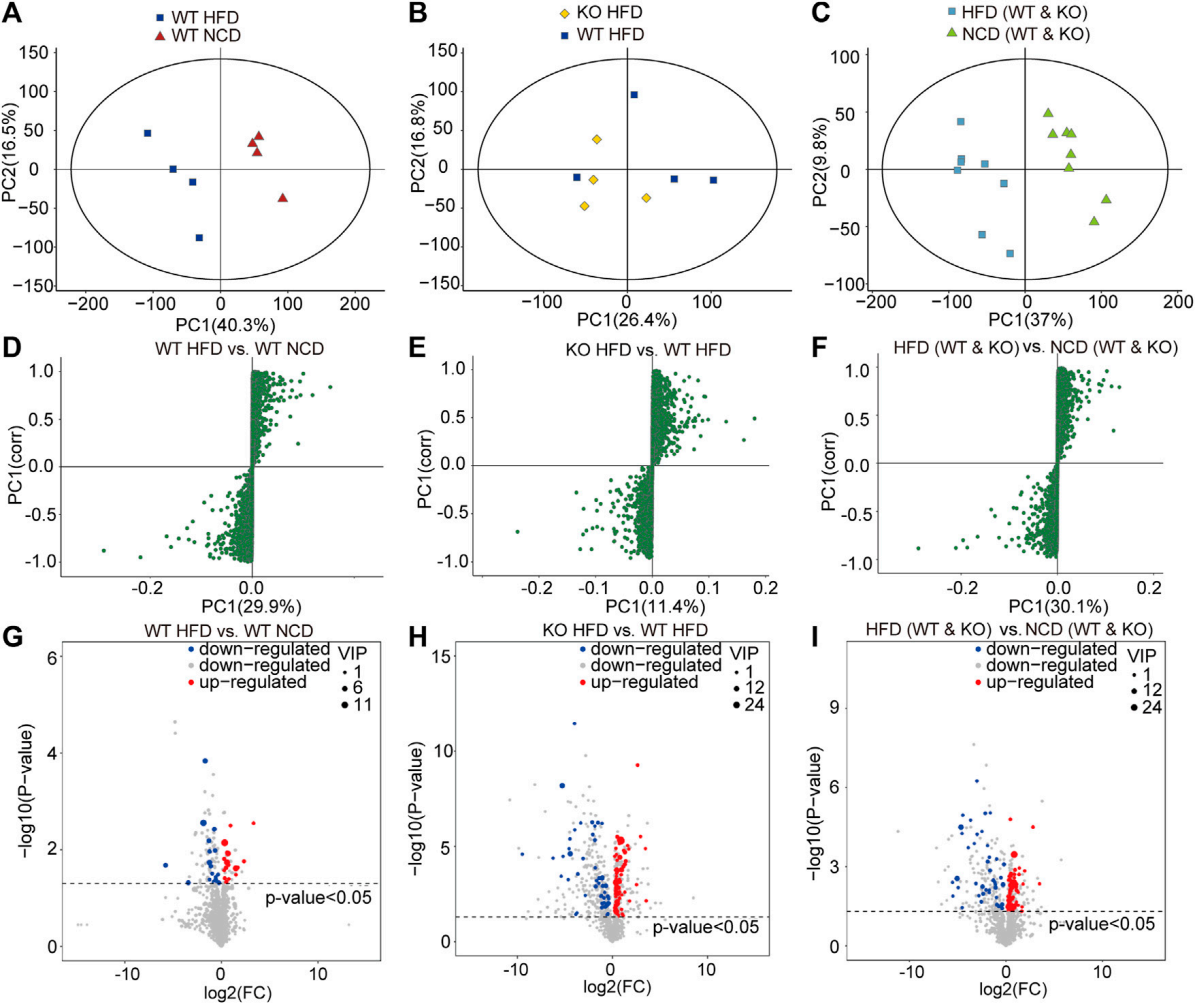
Liver metabolites were measured by metabolomics analysis.**(A)** PCA on species abundances of collembola in WT-HFD and WT-NCD mice. PCA 1 and 2 explain 56.8% of the variation (16.5 and 40.3%, respectively).**(B)** PCA on species abundances of collembola in CD1d KO-HFD and WT-HFD mice. PCA 1 and 2 explain 43.2% of the variation (16.8 and 26.4%, respectively).**(C)** PCA on species abundances of collembola in WT and CD1d KO mice fed with HFD and NCD. PCA 1 and 2 explain 43.2% of the variation (16.8 and 26.4%, respectively). **(D)** S-plot represented for differential metabolites between WT-HFD and WT-NCD mice. **(E)** The differential metabolites between CD1d KO-HFD and WT-HFD. **(F)** The different metabolites between WT and CD1d KO mice fed with HFD and NCD. **(G)** The VIP and *p*-values identified significantly different metabolites between WT-HFD and WT-NCD mice. **(H)** The volcano plot indicated significantly different metabolites between WT-HFD and WT-NCD mice (The screening criteria were VIP value > 1 for the first principal component of orthogonal partial least squares discriminant analysis model and T-test *p*-value <0.05.) **(I)** The volcano plot indicated significantly different metabolites between CD1d KO-HFD and WT-HFD mice. (J) The volcano plot indicated significantly different metabolites between HFD and NCD mice. HFD, high-fat diet; KO, knockout; NCD, normal control diet; PCA, principal component analysis; VIP, variable importance of projection WT, wild-type.

**FIGURE 5:**
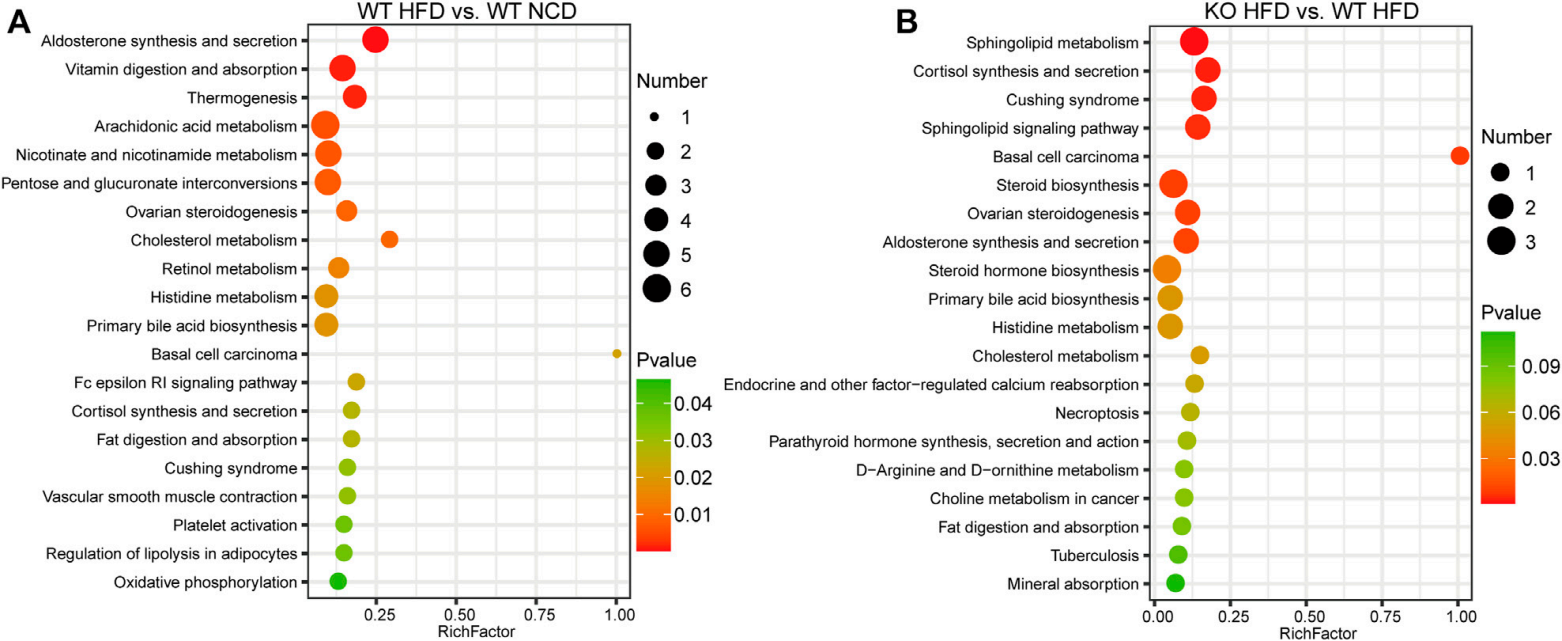
Signal pathways enrichment analysis. **(A)** KEGG analysis indicated the top 20 enriched pathways between WT-HFD and WT-NCD mice. **(B)**The top 20 enriched pathways between CD1d KO-HFD and WT-HFD mice. HFD, high-fat diet; KEGG, Kyoto Encyclopedia of Genes and Genomes; KO, knockout; NCD, normal control diet; WT, wild-type.

To screen and characterize the discriminatory metabolites, we performed principal component analysis (PCA) analysis, hierarchical clustering, and correlation analysis. The quality control (QC) samples are closely clustered in the PCA model diagram through 7-fold cross-validation, which indicated the instrument was stable during the experiment (Supplementary Figure S2A). Examining the spectral intensity of the metabolites revealed the spectrum intensity varied between subgroups (Supplementary Figure S2B). Additionally, the metabolites were significantly different between different subgroups. The orthogonal partial least squares discriminant analysis (OPLS-DA) score plot revealed a clear and distinct clustering between the WT-HFD and WT-NCD groups, the CD1d KO-HFD and WT-HFD groups, and the HFD and NCD groups (Figures 4A–C). The S-plot generated from OPLS-DA revealed significant differences in metabolites between HFD mice and NCD mice. In addition, the CD1d KO-HFD group showed different metabolites compared with the WT-HFD group (Figures 4D–F). To better evaluate the differential metabolites between two subgroups, we constructed volcano plots to visualize *p*-values and fold change values. We examined the *p*-value, variable importance of projection (VIP) value, and fold change value from the volcano plots, which showed remarkable differential metabolites between groups, with red representing significantly upregulated metabolites, blue representing significantly downregulated metabolites, and gray representing insignificant metabolites. We performed hierarchical clustering for the expression levels of the top significant differential metabolites (Figures 4G–I). There were 120 differential metabolites between the WT-HFD and WT-NCD groups, 94 between the CD1d KO-HFD and CD1d KO-NCD groups, 36 between the CD1d KO-HFD and WT-HFD groups, 28 between the WT-NCD and CD1d KO-NCD groups, and 141 between the CD1d KO-HFD and CD1d KO-NCD groups (Table 1). The WT-HFD metabolites revealed several enriched biological processes, including aldosterone synthesis and secretion, vitamin digestion and absorption, thermogenesis, arachidonic acid metabolism, and nicotinate and nicotinamide metabolism compared with the WT-NCD group (Figure 5A). In NAFLD mice, the CD1d KO-HFD group was enriched in the sphingolipid signaling pathway, cortisol synthesis and secretion, Cushing syndrome, sphingolipid metabolism pathways, steroid biosynthesis, ovarian steroidogenesis, and aldosterone synthesis and secretion compared with the WT-HFD group (Figure 5B). To explore correlations between significantly different metabolites, we conducted correlation analysis and metabolism-associated signal pathway enrichment analysis. The results revealed the top 50 differential metabolites in the WT-NAFLD and WT-NCD groups had marked correlations (Supplementary Figure S3). In addition, metabolites in the WT-HFD and CD1d KO-HFD groups were also closely correlated (Supplementary Figure S4). These results demonstrated CD1d plays an important role in metabolic biological processes and signaling pathways in NAFLD progression, which may be useful to help identify clinical interventions for the treatment of hepatic steatosis.

**TABLE 1:** Differential metabolites in all comparison’s groups.

Comparisons	Differential Metabolites
WT-HFD *vs*. WT-NCD	120
CD1d KO-HFD *vs*. CD1d KO-NCD	94
CD1d KO-HFD *vs*. WT-HFD	36
WT-NCD *vs*. CD1d KO -NCD	28
HFD (WT and CD1d KO) *vs*. NCD (WT and CD1d KO)	141

### Integrated Differentially Expressed Metabolites and Genes

**FIGURE 6:**
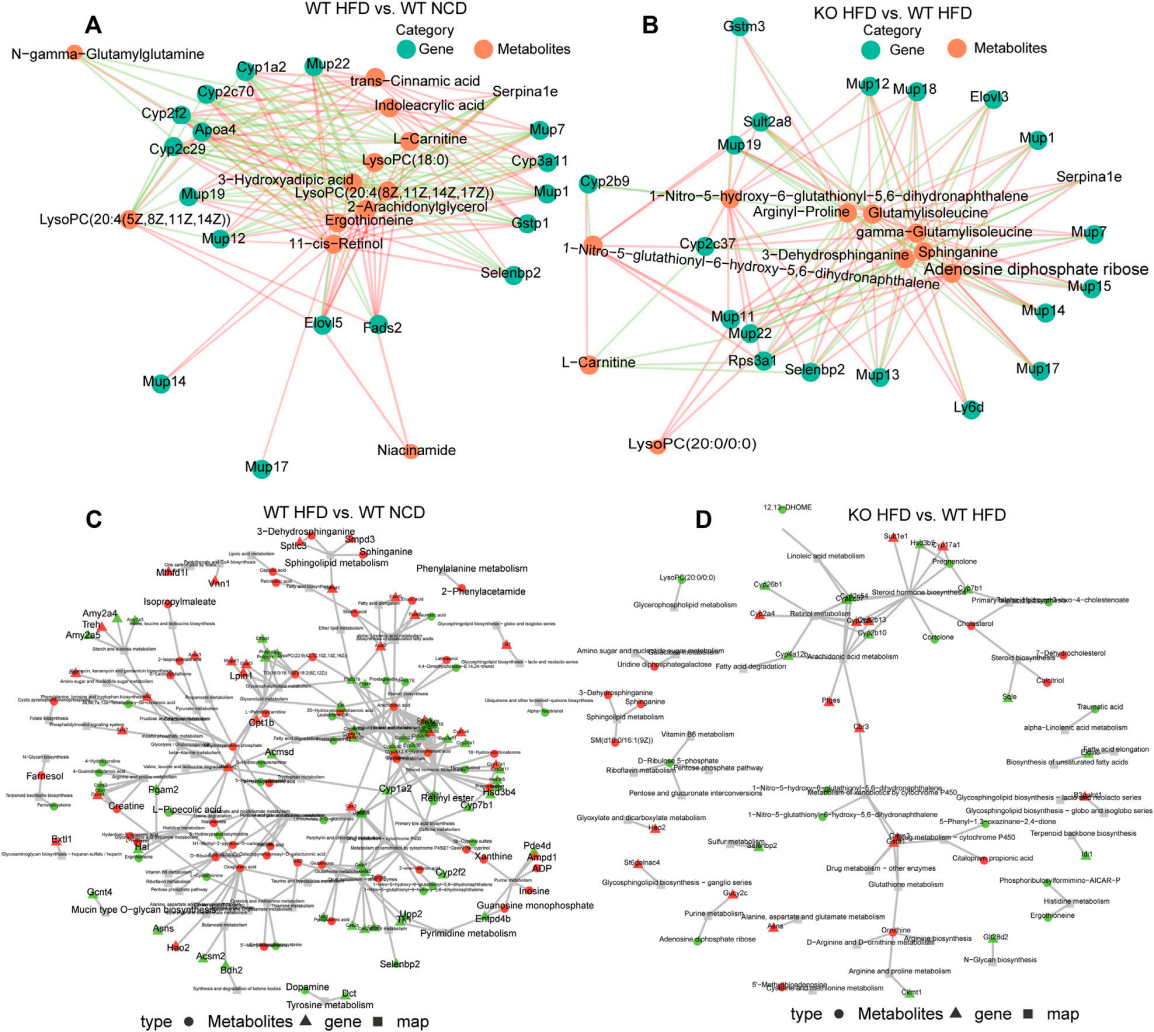
Differently expressed genes and differential metabolites network. **(A)** Network diagram of expression correlation between differential genes and differential metabolites between WT-HFD and WT-NCD mice. The figure shows the network diagram based on Pearson’s correlation analysis to calculate the correlation between the response intensity data of genes and metabolites, and the relationship pairs with *p*-value ≤ 0.05 were selected. The red line represents a positive correlation, and the green line represents a negative correlation. The thickness of the line represents the correlation coefficient. **(B)** Network diagram of expression correlation between differential genes and differential metabolites between CD1d KO-HFD and WT-HFD mice. **(C)** Differentially expressed genes and metabolite-based knowledge-guided machine learning (KGML) network diagram of WT-HFD and WT-NCD groups. Triangles represent genes, circles represent metabolites, and squares represent pathway names. Red is upregulated genes or metabolites, and green is downregulated genes or metabolites. **(D)** Differentially expressed genes and metabolites based on the KGML network diagram of the CD1d KO-HFD and WT-HFD groups. HFD, high-fat diet; KO, knockout; NCD, normal control diet; WT, wild-type.

To better understand the correlation between DEGs and differential metabolites, we integrated the correlation network using a network diagram of expression correlation between differential genes and differential metabolites. Indoleacrylic acid, L-carnitine, trans−cinnamic acid, 3−hydroxyadipic acid, 2−arachidonylglycerol, and ergothioneine have cross relationships with *Mup1*, *Mup12*, *Mup14*, *Mup17*, *Mup19*, *Mup22*, *Mup7*, *Apoa4*, *Cyp1a2*, *Cyp2c29*, *Cyp2c70*, *Cyp2f2*, *Cyp3a11*, *Elovl5*, *Ergothioneine*, *Fads2*, and *Gstp1* (Figure 6A). The network diagram of expression correlation between differential genes and differential metabolites between the CD1d KO-HFD and WT-HFD groups demonstrated remarkable different. The differential metabolites, such as 1−Nitro−5−hydroxy−6−glutathionyl−5,6−dihydronaphthalene; arginyl−proline; gamma−glutamyl isoleucine; glutamyl isoleucine; 3−dehydrosphinganine; adenosine diphosphate ribose; L−carnitine; 3−dehydrosphinganine; and lyso phosphatidylcholine (20:0/0:0), and differential genes, such as *Gstm3*, *Cyp2b9*, *Cyp2c37*, *Elovl3*, *Mup11*, *Mup22*, *Mup13*, *Mup17*, *Mup14*, *Mup15*, *Mup7*, *Mup1*, *Mup18*, *Mup19*, *sult2a8*, *rps3a1*, *selenbp2*, and *ly6D* (Figure 6B). To describe the interactions between transcriptomes and metabolomes systematically and comprehensively, we adopted KEGG analysis and plotted the network relationship between genes and metabolites (Figures 6C,D).

### Intestinal Flora Diversity Analysis

**FIGURE 7:**
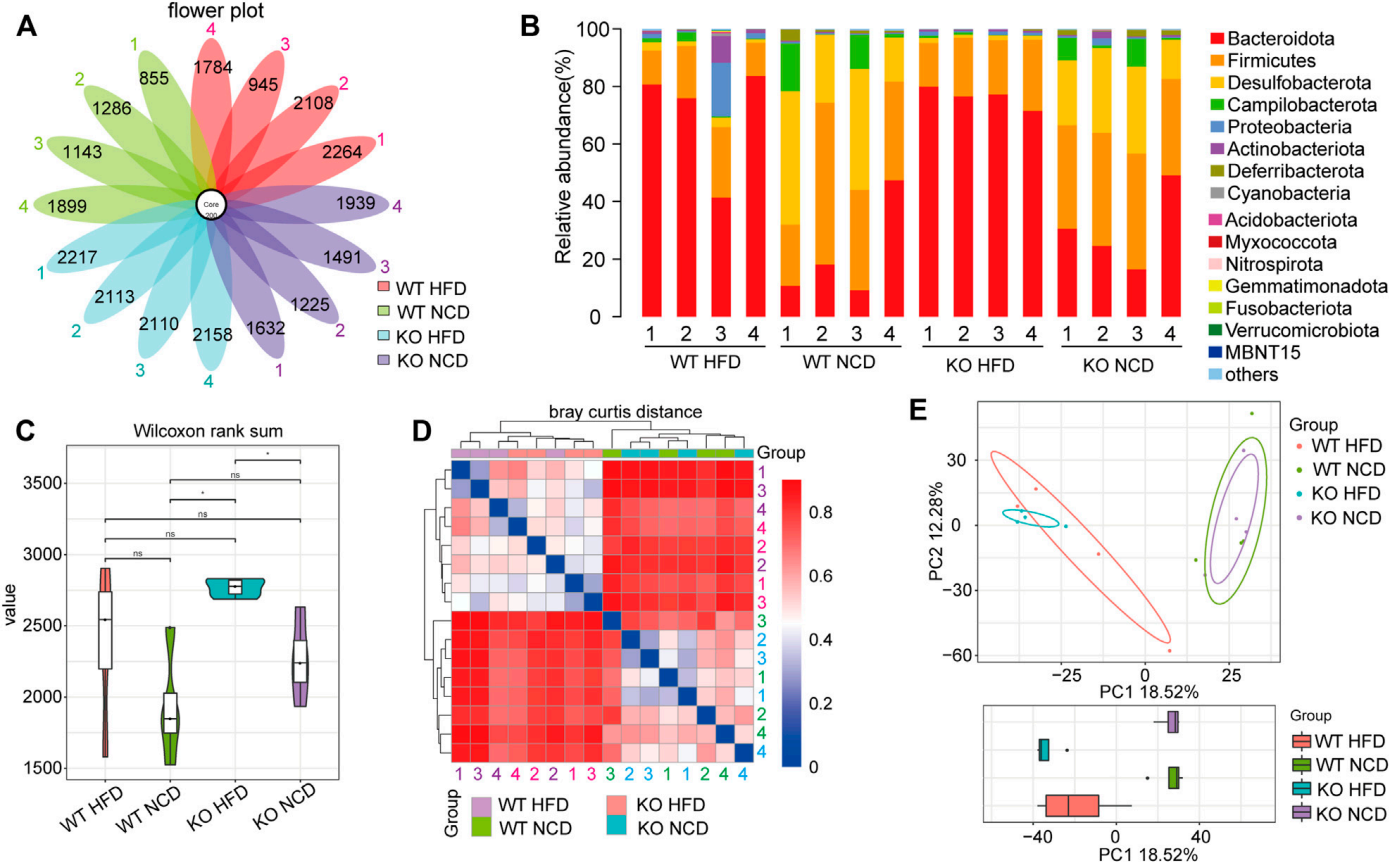
Microbiome analyses were performed using cecal samples. **(A)** Venn diagram showing the distribution of operational taxonomic units (OTUs). The numbers in the center represent the OTUs common to all samples (i.e., Core OTUs), and the numbers on the petals represent the total OTUs of each sample minus the number of OTUs common to all samples. **(B)** The microbial community structure in each sample. The relative abundance is colored in shades of yellow (low relative abundance) to red (high relative abundance). **(C)** Alpha diversity analysis among groups. **(D)** Beta diversity index heatmap in four groups. **(E)** Principal component analysis (PCA) 2D-plot of individuals showing the difference among multiple groups.

To deeply investigate whether CD1d was associated with changes in the fecal microbiota in NAFLD, we performed Illumina MiSeq high-throughput sequencing to explore intestinal flora diversity in fecal samples. The OTUs Venn diagram showed 200 OTUs commonly shared by WT-NCD, CD1d KO-NCD mice, HFD of WT and CD1d KO mice four subgroups. (Figure 7A). The information for each group regarding species, genus, family, order, class, phylum, and kingdom level of OTUs for each sample is shown in Figure 7B. Analysis of the microbial community structure distribution showed the top ten main phyla were Bacteroidetes, Firmicutes, Desulfobacterota, Campylobacteria, Proteobacteria, Actinobacteria, Deferribacterota, Cyanobacteria, Acidobacteriota, and Myxococcota (Figure 7B). CD1d had no remarkable effects on fecal microbiota in NAFLD, as revealed by violin plots showing whole-tree phylogenetic alpha diversity. CD1d KO-HFD mice had a lower alpha diversity value than CD1d-KO-NCD mice. There was a trend toward lower diversity values in NAFLD groups compared with NCD groups (WT NCD group and CD1d KO NCD group) (Figure 7C). We applied beta diversity to deeply explore the microbial diversity among the different groups, and the results indicated the composition of microbial communities of different components was significantly different between the NCD and HFD groups (Figure 7D). We conducted PCA analysis between the four groups (Figure 7E), which illustrated that HFD had a significant effect on the microbiota of different groups. The evolutionary branch diagram of differential bacterial communities or species is presented in Supplementary Figure S5A. We examined the enriched bacterial species in the WT-HFD, which included Muribaculaceae, Anaerofustaceae, Eubacteriales, and Burkholderiales. Additionally, Desulfovibrionaceae, Desulfovibrionales, Desulfovibrio, Oscillospiraceae, Oscillospirales, and Clostridia. In the CD1d KO-HFD group, Lachnospiraceae, Lachnospirales, and Firmicutes were highly abundant (Supplementary Figure S5A). To understand and evaluate the difference in microbial diversity, we conducted the linear discriminant analysis effect size (LEfSe) analysis. The LEfSe results reflected the remarkably distinct microflora between the two groups, and the sample points for each group were relatively close (Supplementary Figure S5B). These results suggest CD1d deficiency might affect intestinal microbial flora during NAFLD progression.

## Discussion

NAFLD has become the most common metabolic disorder, which affects over one-quarter of the global population (Fiorucci and Distrutti, 2021). NAFLD is characterized as a multifactorial, highly complex, and chronic disease that results in aberrant lipid accumulation in hepatocytes (Lim et al., 2021; Masoodi et al., 2021). A growing body of research has demonstrated alterations in metabolism, including metabolic processes, small molecular biochemistry, and membrane transport, could potentially affect NAFLD progression (Masoodi et al., 2021; Nimer et al., 2021). Additionally, potential NAFLD metabolic biomarkers have been identified, including circulating fatty acids, triglycerides, phospholipids, and bile acids.

CD1d is widely expressed on hematopoietic and non-hematopoietic cells (Nimer et al., 2021). Recent studies have suggested CD1d is involved in the pathogenesis of the innate immune responses, lipid metabolism, and microbiota (Maricic et al., 2018; Marrero et al., 2018; Cui et al., 2020). The antigen-presenting cells present pathogen-derived lipid antigens to NKT cells and serve as a link between the innate and adaptive immune systems (Almeida et al., 2019). Notably, NKT cells are activated in a CD1d-dependent manner in the pathological process (Tatituri et al., 2013). Indeed, there is growing evidence suggesting intestinal microbiota significantly impacts NKT cell biology in a CD1d-dependent manner (Tatituri et al., 2013). The representative microbial and endogenous NKT cell antigens include glycolipids, glycosphingolipids, diacylglycerols, glycerophospholipids, lysophospholipids, and cholesterol esters (Mattner, 2013). In this study, we revealed CD1d-deficient mice fed with an HFD for 24 weeks had increased body weight gain, lipid accumulation, and hepatic steatosis compared to WT mice fed with an HFD. We hypothesized that CD1d deficiency not only affects the HFD-induced NAFLD immune microenvironment but is also involved in NAFLD-associated metabolite and microbial diversity. In this study, we comprehensively analyzed the transcriptomes, metabolomes, and gut microbiotas in WT mice and CD1d KO mice fed with HFD or NCD. NAFLD is a metabolism-associated chronic disease, which is characterized by both triglyceride and free cholesterol accumulation (Qian et al., 2021). Indeed, dysregulated cholesterol metabolism might contribute to NAFLD progression (Hu et al., 2021). The pathological and biological regulation of NAFLD is complex, and transcriptomic signatures suggest hepatic lipids may play key functions in NAFLD initiation and progression (Hu et al., 2021). In this analysis we figured out that the CD1d might play protective role in hepatic steatosis stage. Based on transcriptomic analysis. We also found out that the sphingolipid metabolism signal pathways, cortisol synthesis and secretion pathway, Cushing syndrome, sphingolipid signal pathway, steroid biosynthesis, ovarian steroidogenesis et al. signal pathways, which closely correlated with lipid accumulation and hepatic steatosis. Sphingolipids are a group of bioactive lipids which regulate multiple lipid metabolism pathways and programs relevant to the development of NAFLD (Montefusco et al., 2022). The steroid biosynthesis was also involved in lipid accumulation and hepatic steatosis (Yanai et al., 2022). However, the relationship between CD1d and sphingolipid metabolism and steroid biosynthesis process has not been reported.

Furthermore, liver-gut microbiota are key regulators responsible for maintaining liver homeostasis (Lee et al., 2020; Rom et al., 2020). Consequently, disturbances in gut microbiota (dysbiosis) are also associated with NAFLD (Chen et al., 2019). The hepatocyte-expressed CD1d, which presents microbiota lipid antigen, is also involved in NAFLD progression (Wang and Gao, 2020). Li et al. uncovered that the gut commensal microbes are involved in NAFLD through CD1d/lipid antigens presentation to liver-resident γδT-17 cells (Wang and Gao, 2020).

In the present study, we identified differentially enriched signaling pathways, altered liver metabolites, and the most dominant bacterial groups between WT-HFD mice and CD1d-deficient mice. The signal pathways have been reported play important role in lipid accumulation and liver steatosis. The involvement of CD1d gene in metabolism associated signaling has not been previously demonstrated. The regulation of CD1d and its exact mechanism in NAFLD progression remain unknown. Nonetheless, our results indicate several transcriptomic, metabolomic, and gut microbiota-associated mechanisms through which CD1d contributes to NAFLD progression.

## Conclusion

In summary, our results provide critical insights into the relationship between CD1d KO and NAFLD progression through analysis and integration of transcriptomics, metabolic profiles, and gut microbiomics.

## Data Availability

The datasets presented in this study can be found in online repositories. The names of the repository/repositories and accession number(s) can be found in the article/Supplementary Material.

## Supplementary Material

Supplementary Figure S1 Signal pathway enrichment analysis *via* KEGG. **(A)** The top 20 pathways enriched in KEGG between WT-HFD and WT-NCD mice. **(B)** The top 20 pathways enriched in KEGG between CD1d KO-HFD and WT-HFD mice.

Supplementary Figure S2 QC quality control. **(A)** The principal component analysis (PCA) score plots for all samples. **(B)** The evaluation of metabolite strength distribution of quality control (QC) samples.

Supplementary Figure S3 The correlations of differential metabolites in WT-HFD and WT-NCD.

Supplementary Figure S4 The correlations of differential metabolites in CD1d KO-HFD and WT-HFD.

Supplementary Figure S5 LEfSe analysis in different comparisons. **(A)** Differences in species abundance between four groups. **(B)** Differences in species annotation branch diagram. Different colors represent different groups. Significant differences were found in relatively high abundance species annotated in corresponding color nodes.
