# Supplementary figures and images for: Multi-Omics Characterizes the Effects and Mechanisms of CD1d in Nonalcoholic Fatty Liver Disease Development
Source: Front Cell Dev Biol. 2022 Apr 8;10:830702. doi: 10.3389/fcell.2022.830702 (PMC9024148; doi:10.3389/fcell.2022.830702)

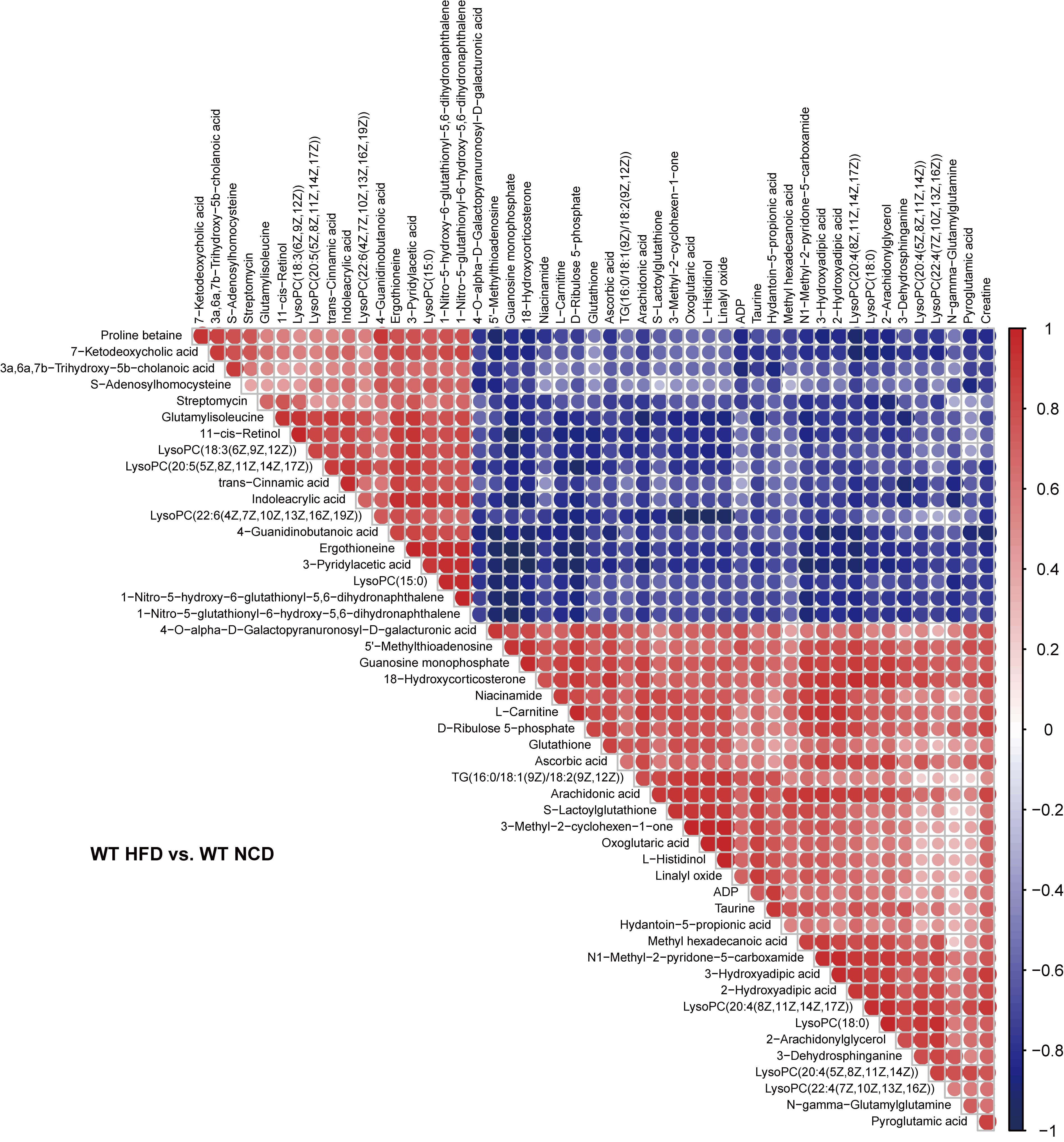

Supplement: Supplementary file 1 [file Image3.TIF]

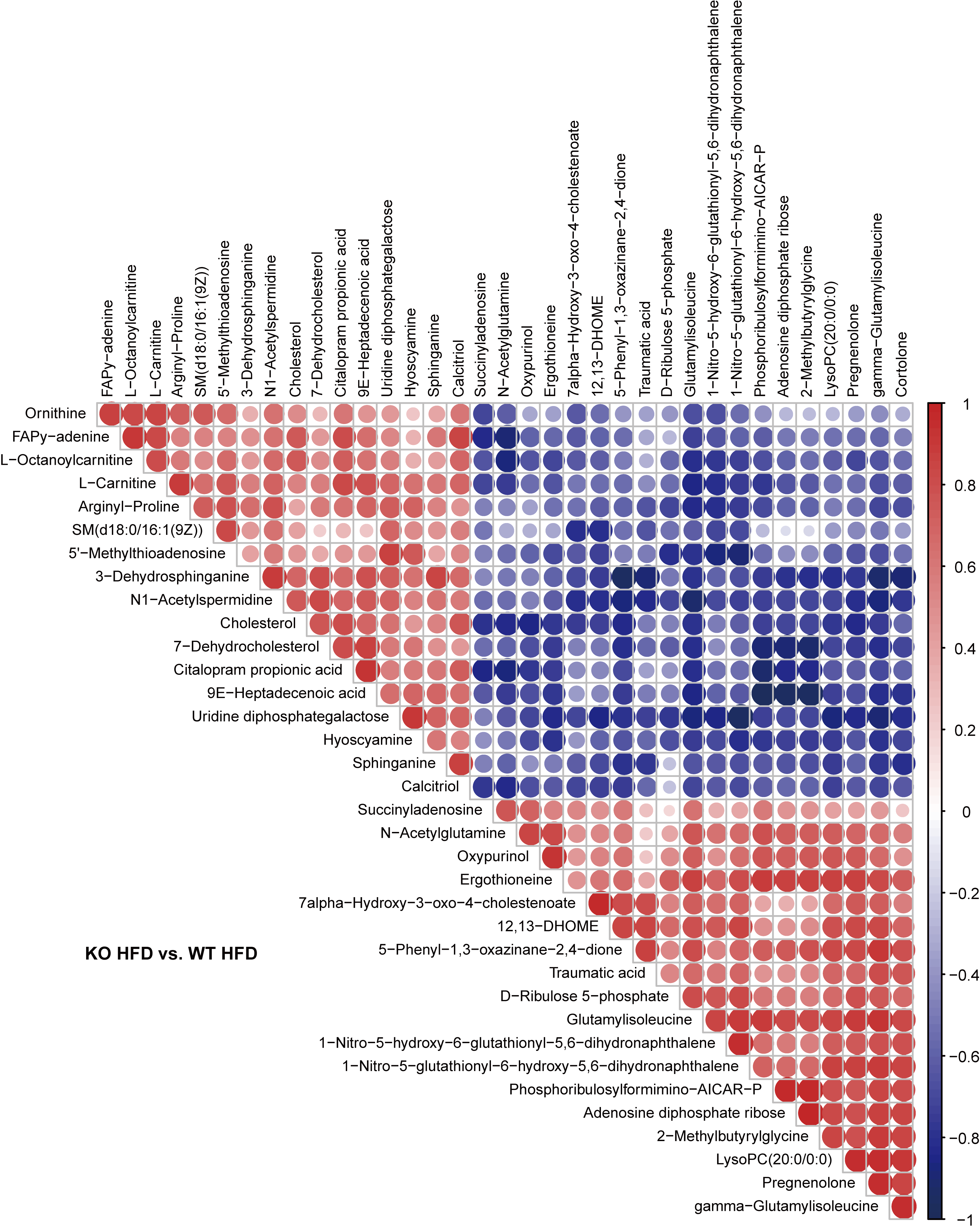

Supplement: Supplementary file 2 [file Image4.TIF]

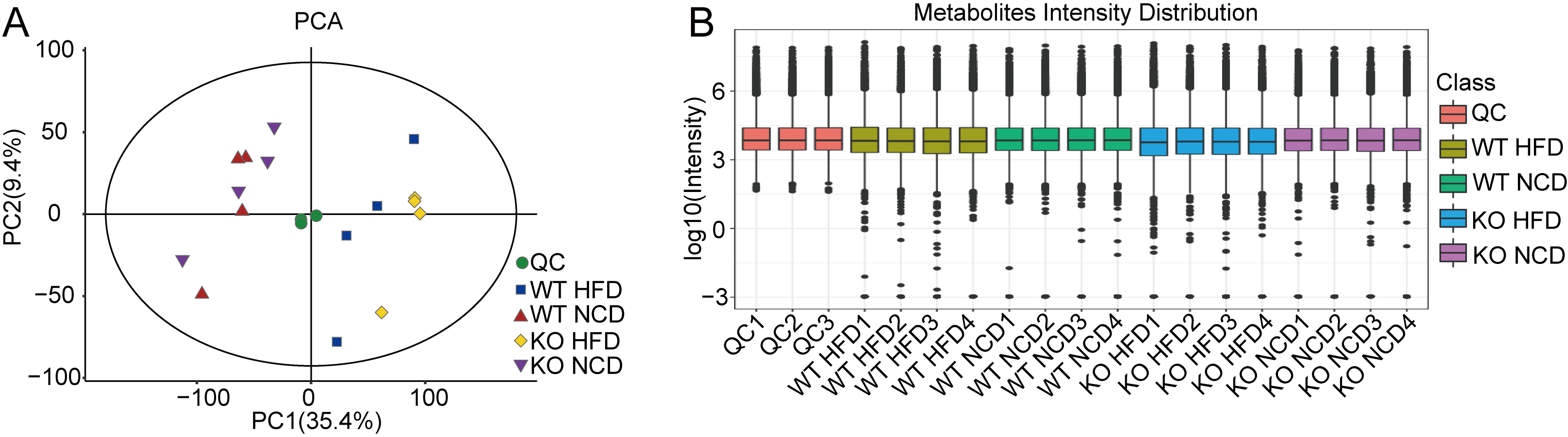

Supplement: Supplementary file 3 [file Image2.TIF]

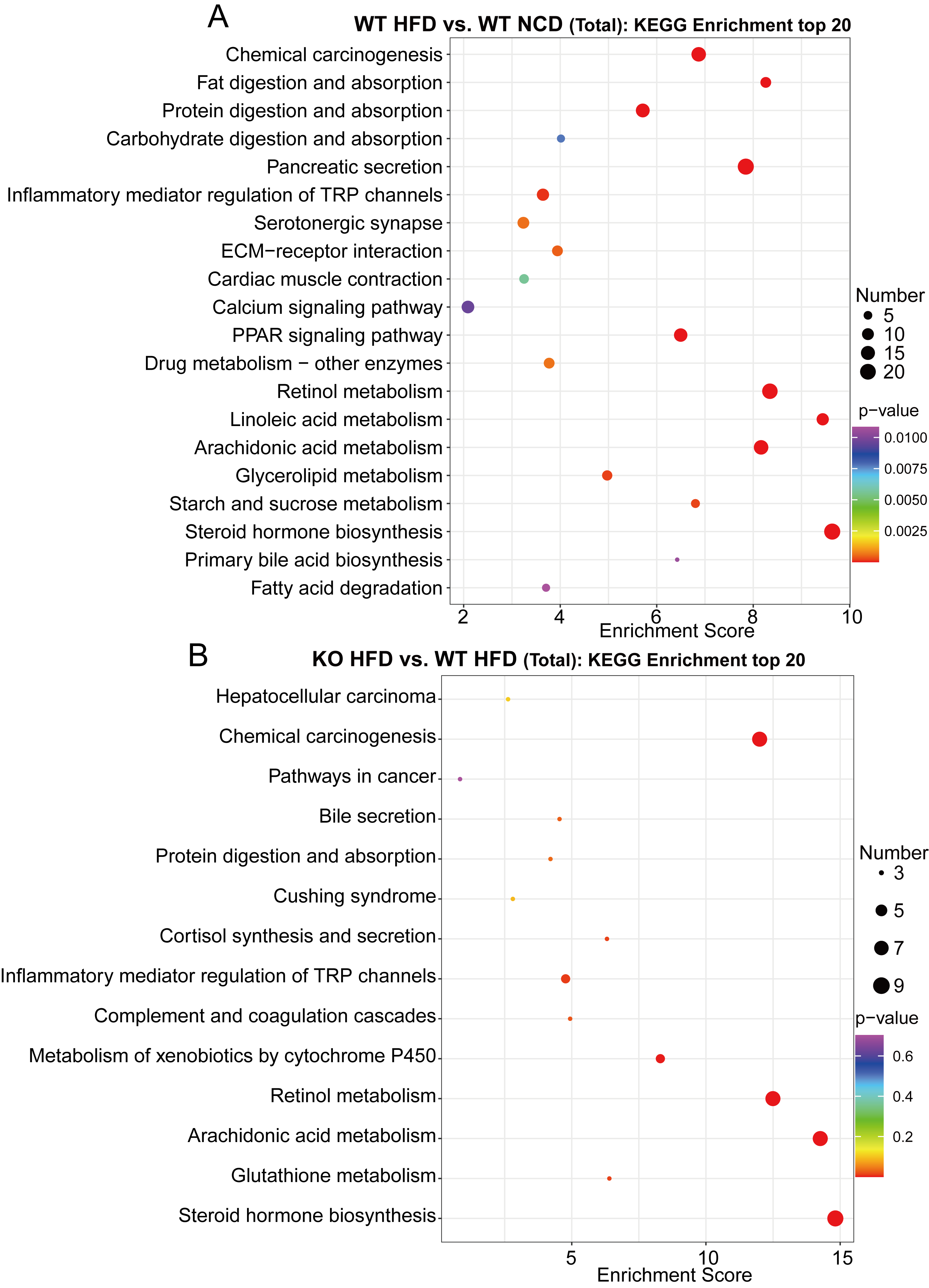

Supplement: Supplementary file 4 [file Image1.TIF]

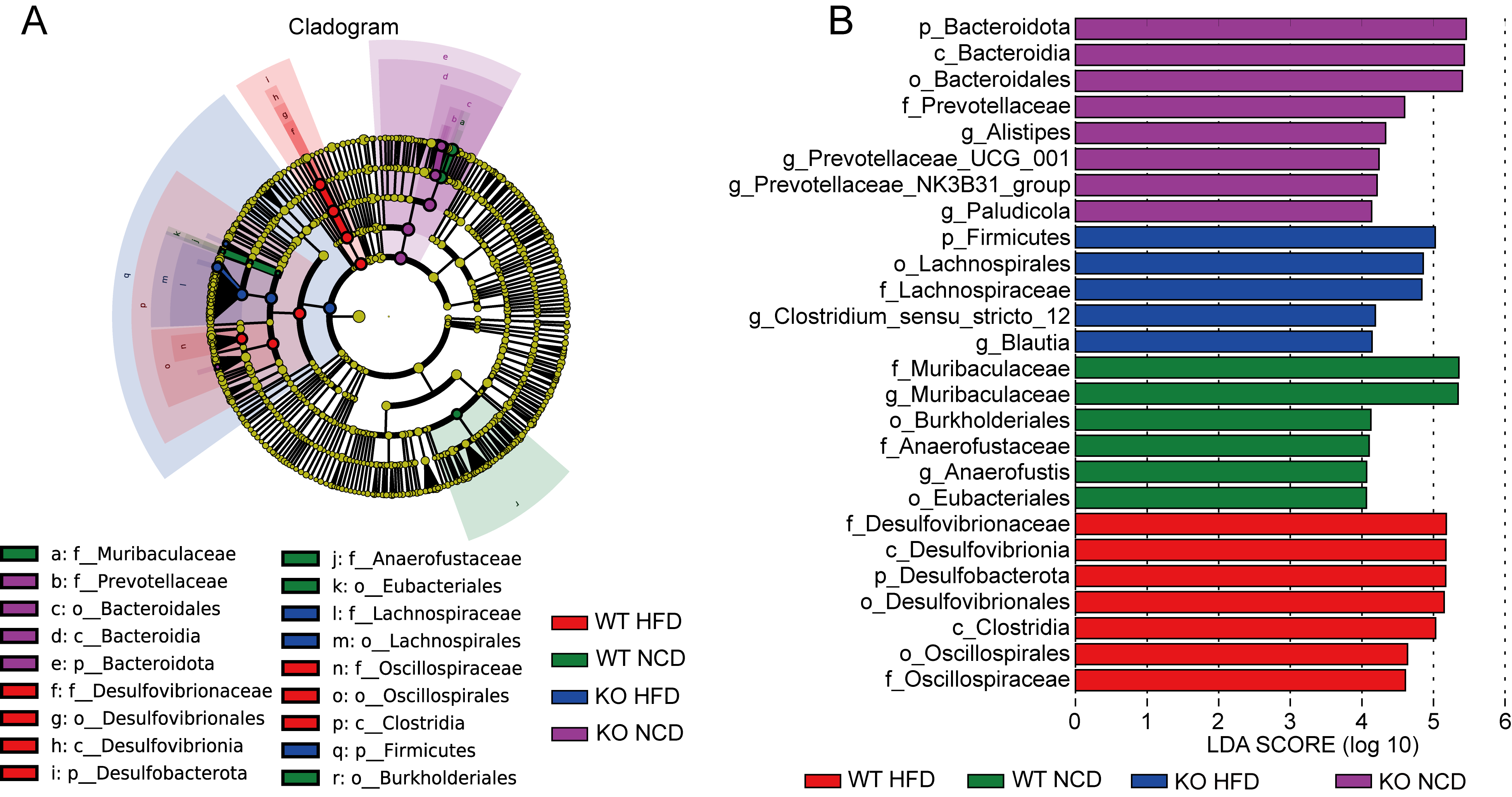

Supplement: Supplementary file 5 [file Image5.TIF]
